# 甲状腺转录因子在宣威肺腺癌中的表达及临床意义

**DOI:** 10.3779/j.issn.1009-3419.2013.03.03

**Published:** 2013-03-20

**Authors:** 云芬 俎, 熙才 王, 馨 刘, 艳 陈, 承文 李, 学琴 尚, 余澄 谢, 虹园 赵, 继营 王

**Affiliations:** 1 650021 昆明，云南省第二人民医院肿瘤科 Department of Oncology, the Second People Hospital of Yunnan Province, Kunming 650021, China; 2 650118 昆明，云南省肿瘤医院肿瘤研究所 Yunnan Tumor Institute, the Third Affiliated Hospital, Kunming 650118, China; 3 650118 昆明，云南省肿瘤医院科研科 Department of Research Management, the Third Affiliated Hospital, Kunming 650118, China; 4 650021 昆明，云南省第二人民医院病理科 Departments of Pathology, the Second People Hospital of Yunnan Province, Kunming 650021, China; 5 650021 昆明，昆明市盘龙区人民医院急诊科 Department of Emergency Medicine, People Hospital of Panlong District, Kunming 650021, China

**Keywords:** 甲状腺转录因子, Ki-67, 肺肿瘤, 预后, TTF-1, Ki-67, Xuanwei lung adenocarcinoma line, Xuanwei lung adenocarcinoma, Prognosis

## Abstract

**背景与目的:**

通过检测宣威肺腺癌细胞株（XWLC-05）中甲状腺转录因子（thyroid transcription factor 1, TTF-1）mRNA和蛋白表达及宣威肺腺癌标本组织TTF-1和Ki-67蛋白的表达，分析宣威肺腺癌中TTF-1与Ki-67表达相关性及对患者预后的影响。

**方法:**

利用qRT-PCR方法检测XWLC-05中TTF-1 mRNA和免疫组化分析TTF-1及Ki-67蛋白表达。同时收集2008年1月-2012年3月96例手术后宣威肺腺癌组织标本，免疫组化检测TTF-1及Ki-67的蛋白表达。

**结果:**

XWLC-05中TTF-1 mRNA低表达，免疫组化TTF-1表达阴性，Ki-67 100%表达。96例肺癌患者TNM分期Ⅰ期-Ⅱ期66%（63/96）、Ⅲ期34%（33/96），TTF-1蛋白表达率93%（89/96），Ki-67为70%（67/96)，其中高分化腺癌TTF-1阳性表达率98%（31/39)，中低分化腺癌89%（51/57)。TTF-1阳性表达随着分化程度的增加而增高（*P*=0.002），与Ki-67的表达负相关（*P*=0.01），与性别、年龄、吸烟史及分期无关。*Kaplan*-*Meier*生存曲线显示患者中位无进展生存（median progression-free survival, PFS）与分期、TTF-1及Ki-67表达强度有关，Ⅰ期-Ⅱ期PFS高于Ⅲ期（46个月*vs* 32个月，*P*=0.001），TTF-1强阳性高于弱阳性和阴性组（45个月*vs* 35个月，*P*=0.036），Ki-67弱阳性和阴性高于强阳性组（46个月*vs* 40个月，*P*=0.048）。

**结论:**

TTF-1在肺腺癌中可能是一个肿瘤抑制基因，基于它能抑制Ki-67的表达，并且可作为宣威肺腺癌好的预后指标。

## Abstract (en)

**Background and objective:**

The present study aims to detect the expressions of the thyroid transcription factor 1 (TTF-1) mRNA and protein in Xuanwei lung adenocarcinoma line (XWLC-05). We also investigated the relationship of TTF-1 with Ki-67 protein and analyzed the prognostic value of the TTF-1 protein in Xuanwei lung adenocarcinoma patients.

**Methods:**

The expression of TTF-1 mRNA was evaluated by quantitative real-time RT-PCR (qRT-PCR), while proteins of TTF-1 and Ki-67 were detected by immunohistochemistry in XWLC-05. The expressions of TTF-1 and Ki-67 was detected in 96 resected cases of Xuanwei lung adenocarcinoma by immunohistochemistry from January 2008 to March 2012.

**Results:**

TTF-1 mRNA was low and protein was absent in XWLC-05. The expression of Ki67 protein was 100% (high proliferative activity) in XWLC-05. All samples were Xuanwei lung adenocarcinoma (*n*=96) and TNM stages were Ⅰ-Ⅱ 66% (63/96) and Ⅲ 34% (33/96). Immunohistochemical analysis showed the expression of TTF-1 in 89 (93%) of 96 and Ki-67 in 69 (70%) of 96. The positive rate of TTF-1 protein was 38 (96%) of 39 in well-differentiated phenotype and 51 (89%) 57 in moderately/poorly-differentiated phenotype. Patients with strong immunohistochemical expression of TTF-1 were statistically associated with well-differentiated phenotype (*P*=0.002), has inverse correlation with Ki-67 expression (*P*=0.01), and no correlation with age, sex, smoking history, and stages. The result from the *Kaplan*-*Meier* survival analysis showed that stages TTF-1 and Ki-67 were significantly associated with the prognosis of Xuanwei lung adenocarcinoma patients. The median progression-free survival in patients with Ⅰ-Ⅱ stages, strong positive expression TTF-1 and negative, and weak expressions Ki-67 groups was remarkably higher than those patients with Ⅲ stage (46 months *vs* 32 months, *P*=0.001), negative and weak expressions TTF-1 (45 months *vs* 35 months, *P*=0.036) and strong positive expression Ki-67 groups (46 months *vs* 40 months, *P*=0.048), respectively.

**Conclusion:**

Our results presented here suggest that TTF-1 may serve as a tumor suppressor gene based on its inverse correlation with Ki-67 proliferative activity. Patients with strong TTF-1 protein expression group tend to have a significantly better prognosis than patients with negative and weak TTF-1 protein expression group in Xuanwei lung adenocarcinoma.

肺癌是最常见的恶性肿瘤，死亡率居全世界肿瘤死亡之首^[1]^，80%以上为非小细胞肺癌（non-small cell lung cancer, NSCLC），其中肺腺癌发病率逐渐增加，占NSCLC的50%，成为最常见的组织学类型。云南宣威肺癌以女性腺癌为主，其特点为女性发病率高，死亡高峰年龄提前，室内污染及人群遗传易感性是主要发病原因^[2]^。进一步探索宣威肺癌组织生物学行为和临床特征，判断预后一直是人们关注焦点。

甲状腺转录因子（thyroid transcription factor 1, TTF-1）在II型肺泡细胞和细支气管上皮细胞中表达，并且60%-80%的肺腺癌亦表达TTF-1^[3-5]^。研究表明，TTF-1的表达与肺腺癌患者预后有密切相关。本文通过实时定量PCR及免疫组织化学方法检测TTF-1在XWLC-05中的mRNA及蛋白表达情况及与Ki-67的关系，观察二者在宣威肺腺癌组织中表达的相关性和对预后的影响，旨在评价TTF-1在宣威肺癌的临床应用价值以及对预后影响。

## 1 材料与方法

### 1.1 肺癌细胞株及细胞培养

选取H157细胞株（鳞癌，不表达TTF-1 mRNA）和细胞株MGH24（腺癌，高表达TTF-1 mRNA）^[6]^作为对照，检测细胞株XWLC-05 TTF-1 mRNA表达，XWLC-05^[7]^来源于一宣威女性，右肺中分化腺癌术后标本，经过原代和传代培养，生长稳定，裸鼠成瘤率高。H157、XWLC-05及MGH24三株细胞均来自云南省肿瘤研究所。于5%CO_2_、37 ℃、饱和湿度的培养箱中培养传代，以RPMI-1640培养基（含10%小牛血清）培养，0.25%胰酶-EDTA消化传代。

### 1.2 qRT-PCR检测XWLC-05TTF-1 mRNA的表达

取对数生长期的细胞（H157、XWLC-05及MGH24）用Trizol法提取各组总RNA，核酸蛋白检测仪检测RNA纯度，RNA电泳检测RNA完整性。按照cDNA试剂盒说明书进行逆转录合成cDNA。TTF-1上游引物序列：5'-GAGGGAGGAGCAGCCCC-3'，下游引物序列：5'-CCACTTTCTTGTAGCTTTCCTCCA-3'以β-actin为内参，上游引物序列：5’-ACATCTGCTGGAAGGTGG AC-3’，下游引物序列：5’-GGTACCACCATGTACCCA GG-3’。按SYBGreen试剂盒（Bio-Rad公司）说明书进行扩增，90 ℃变性10 min后，按下述条件扩增40个循环：95 ℃、30 s，60 ℃、60 s，循环40次。mRNA相对表达量=2^-△△Ct^，其中^-△Ct^=（目的基因的平均Ct值-内参基因的平均Ct值）。

### 1.3 XWLC-05细胞免疫组化检测

鼠抗人TTF-1单克隆抗体、兔抗人Ki-67单克隆抗体、免疫组化试剂盒和DAB显色试剂盒均购自福州迈新生物技术开发有限公司，使用即用型（Maxvision^TM^ 2/HRP免疫组化染色，步骤按照产品说明书进行。细胞经过冰丙酮固定，抗体孵育，DAB显色。在镜下观察阳性细胞比例。

### 1.4 标本收集

昆明医科大学附属医院2008年1月-2012年3月手术切除原发性宣威肺癌组织标本96例，所有病例均为病理切片HE染色确诊的原发性肺腺癌。采集病例相关的临床病理资料，包括年龄、性别、吸烟史、分化程度及TNM分期；病理类型根据2004年WHO肺、胸膜肿瘤诊断标准；肿瘤分期按2002年国际抗癌联盟TNM分期标准。

### 1.5 宣威肺癌病例免疫组化检测

96例宣威肺腺癌的病例经再次核对组织病理学诊断。免疫组化SP法检测，主要试剂鼠抗人TTF-1、兔抗人Ki-67单克隆抗体及免疫组化试剂盒均为福州迈新公司产品。切片经常规脱蜡，用3%过氧化氢去除内源性过氧化物酶，高温抗原修复。免疫组化染色方法和步骤严格按试剂盒说明书进行。分别用PBS代替一抗作空白对照，已知阳性（细支气管肺泡癌）切片做阳性对照。每张切片高倍镜下随机选取10个视野共记录1, 000个细胞，使用半定量法计算TTF-1及Ki-67细胞核染数目百分比：0；（+）为 < 25%；（++）为26%-50%；（+++）为>50%。

### 1.6 随访和生存分析

末次随访时间为2012年9月1日。无进展生存期（progression-free survival, PFS）：首次治疗开始至肿瘤复发或进展的时间，最短随访时间为4个月，最长随访时间为4年，有6例失访。

### 1.7 统计学分析

采用SPSS 17.0统计软件，*Wilcoxon*秩和检验和*Spearman*等级相关分析TTF-1及Ki-67阳性率，TTF-1与Ki-67表达相关性，应用*Kaplan*-*Meier*法（组间生存率比较采用*Log*-*rank*检验）进行生存分析。*P* < 0.05为差异有统计学意义。

## 2 结果

### 2.1 TTF-1

mRNA在XWCL-05细胞系中表达在3株细胞中已知H157不表达，而MGH24高表达TTF-1，XWLC-05 TTF-1 mRNA的表达水平与H157比较为0.55，MGH24为1.3，显示XWCL-05低表达TTF-1（图 1）。

**1:**
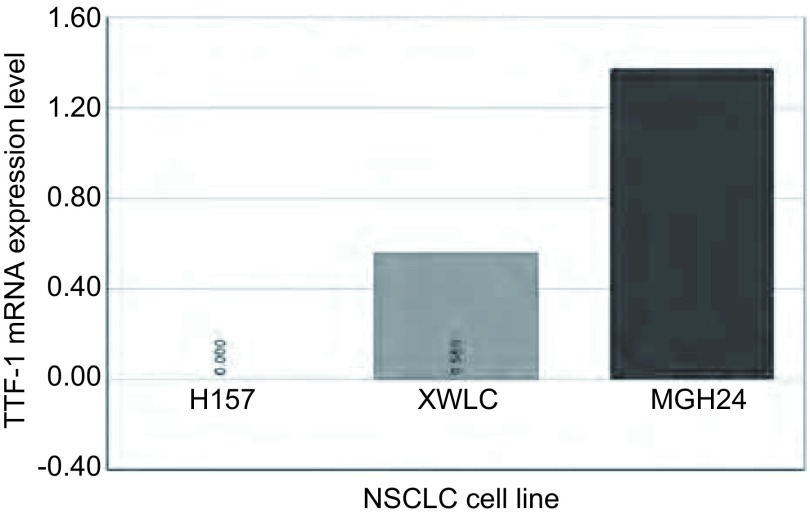
逆转录实时定量PCR检测3个非小细胞肺癌株mRNA表达 The reverse transcription and real-time PCR analysis of mRNA transcript levels in 3 non-small cell lung cancer (NSCLC) cell lines

### 2.2 免疫组化分析TTF-1及Ki-67在XWLC-05中蛋白表达

TTF-1在XWLC-05中表达阴性，Ki-67为100%表达（图 2）。

**2:**
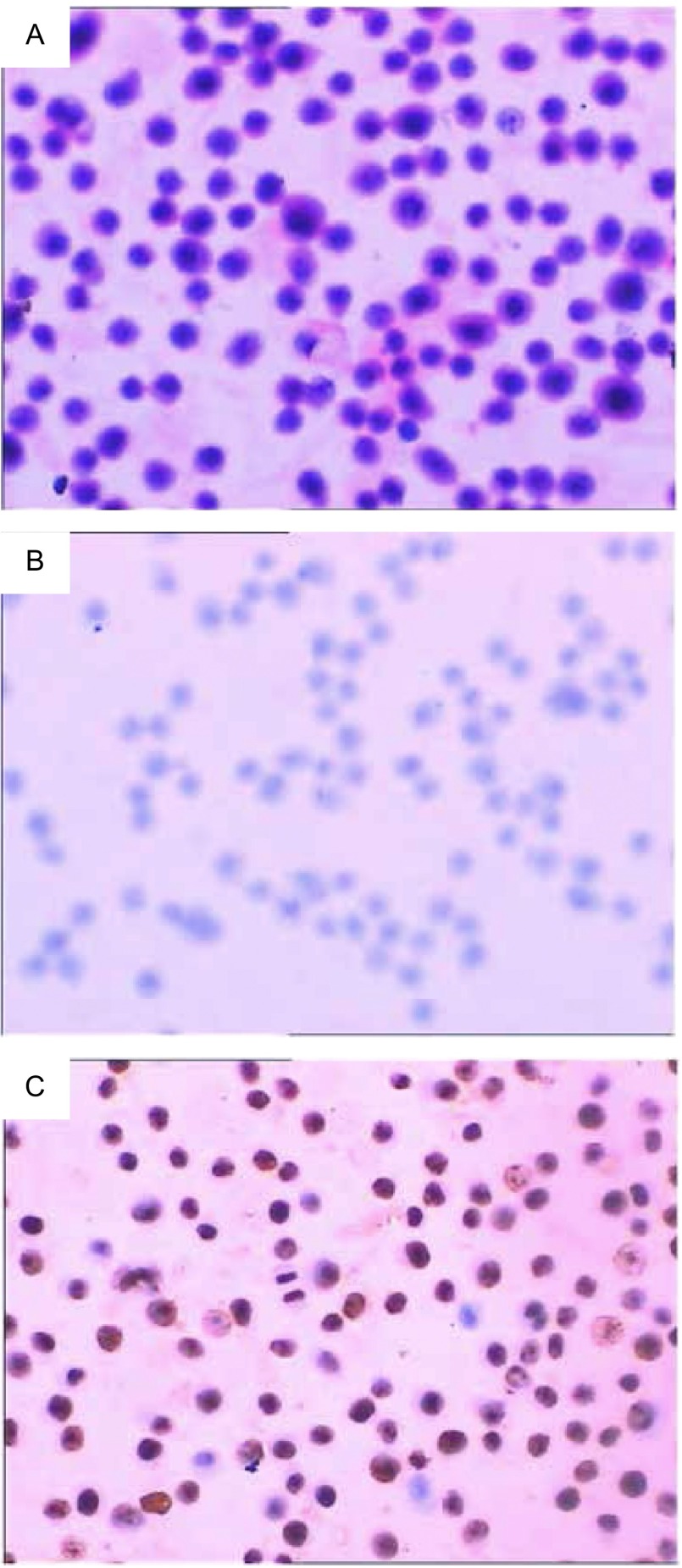
免疫组化分析TTF-1及Ki-67在XWLC细胞中表达。A：XWCL-05 (HE染，×200)，B：TTF-1（-）(IHC，×200)，C：Ki-67（+++）(IHC，×200) Immunohistochemistry for TTF-1 and Ki-67 in Xuanwei lung adenocarcinomas line XWCL-05. A: XWCL-05 (HE, ×200); B: TTF-1(-) (IHC, ×200); C: Ki-67(+++)(IHC, ×200)

### 2.3 临床病例资料

96例宣威肺腺癌患者，其中男性43例，女性53例；平均年龄男性54岁，女性51岁；不吸烟47例，吸烟49例；高分化39例，中低分化57例；Ⅰ期+Ⅱ期63例，Ⅲ期33例。统计学结果显示，TTF-1阳性表达程度随肿瘤分化程度的增加而增高（*P*=0.002），而与性别、年龄、吸烟等无关（*P*>0.05）（表 1，图 3）。

**1:** TTF-1和Ki-67蛋白表达与宣威肺腺癌的临床资料相关性 Correlations between TTF-1 and KI-67 expressions and clinicopathological features in Xuanwei lung adenocarcinomas

Characteristic	*n*	TTF-1				*P*	Ki-67				*P*
		0	(+)	(++)	(+++)		0	(+)	(++)	(+++)	
Gender						0.86					0.90
Male	43	3	11	14	15		13	9	10	11	
Female	53	4	13	16	20		16	10	13	14	
Age (year)						0.84					0.10
< 60	66	5	17	20	24		20	13	16	17	
≥60	30	2	7	10	11		9	6	7	8	
Smoking history						0.88					0.55
Yes	49	4	11	16	18		16	10	11	12	
No	47	3	13	14	17		13	9	12	13	
Differentiation						0.002					0.40
Well	39	1	5	13	20		14	8	7	10	
Moderately/poorly	57	6	19	17	15		15	11	16	15	
TNM stage						0.86					0.029
Ⅰ+Ⅱ	63	4	16	20	23		22	14	15	12	
Ⅲ	33	3	8	10	12		7	5	8	13	

**3:**
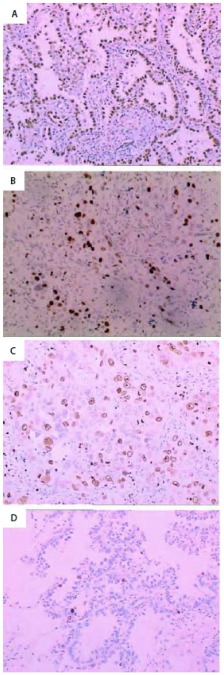
免疫组化检测宣威肺腺癌TTF-1和Ki-67的蛋白表达。A：TTF-1在高分化腺癌表达（+++）（IHC，×100）；B：TTF-1在低分化腺癌表达（+）（IHC，×100）；C：Ki-67在低分化腺癌表达（++）（IHC，×100）；D：Ki-67在高分化腺癌表达（+）（IHC，×100） The TTF-1 and Ki-67 proteins are detected by immunohistochemistry. A: TTF-1 in well differentiation of Xuanwei adenocarcinoma (+++)(IHC, ×100); B: TTF-1 in poorly differentiation of Xuanwei adenocarcinoma (+)(IHC, ×100);C: Ki-67 in poorly differentiation of Xuanwei adenocarcinoma (++)(IHC, ×100); D: Ki-67 in well differentiation of Xuanwei adenocarcinoma (+)(IHC, ×100)

### 2.4 TTF-1和Ki-67在宣威肺癌相关性分析

经过*Spearman*等级相关分析，在96例肺癌组织中TTF-1和Ki-67的表达呈负相关（*P*=0.01）（表 2）。

**2:** 宣威肺腺癌中TTF-1与Ki-67的相关性 Correlation between TTF-1 expression and Ki-67 proliferative activity in Xuanwei lung adenocarcinomas

Ki-67 immunoreactivity	*n*	TTF-1 expression				*P*
		(-)	(+)	(++)	(+++)	
Positive	67	6	21	20	20	0.01
Negative	29	1	3	10	15	

### 2.5 生存分析

*Kaplan*-*Meier*生存曲线显示，TTF-1强阳性组的中位PFS与阴性和弱阳性组比较有统计学意义（45个月*vs* 35个月，*P*=0.036）（图 4A），Ⅰ期-Ⅱ期组与Ⅲ期组中位PFS比较有统计学意义（46个月*vs* 32个月，*P*=0.001）（图 4B），Ki-67阴性和弱阳性组中位PFS与强阳性组比较有统计学意义（46个月*vs* 40个月，*P*=0.048）（图 4C），患者中位PFS与性别、年龄、吸烟及肿瘤分化程度无关。

**4:**
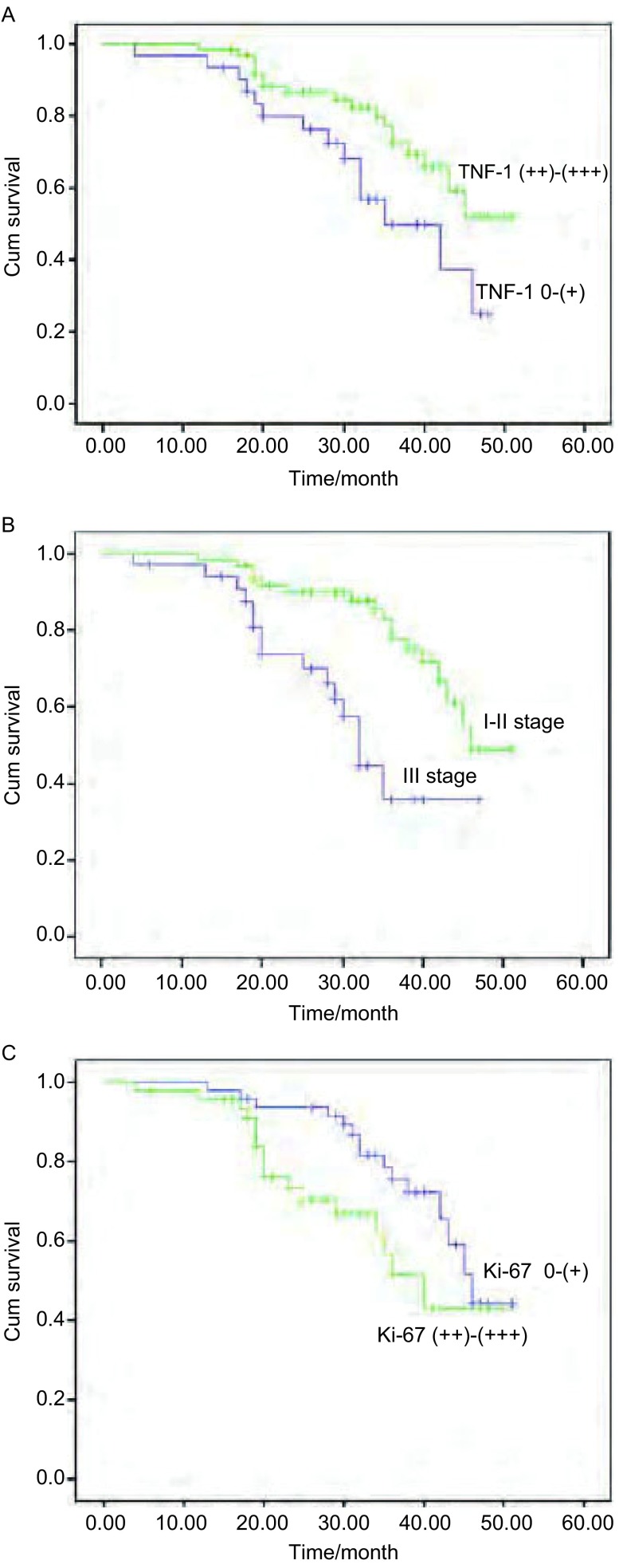
不同因素组96例宣威肺癌患者的中位无进展生存曲线。A：TTF-1强度；B：分期；C：Ki-67强度 Median PFS curves of different variables of 96 patients with Xuanwei lung adenocarcinoma. A: TTF-1; B: stages; C: Ki-67

## 3 讨论

TTF-1是核转录蛋白NKx2基因家族成员之一，是常染色体14q13单一位点基因编码的产物，分子量38 kDa，参与调控许多基因的表达。TTF-1在肺组织形成早期阶段就开始并且终身表达。动物实验^[8]^表明，*TTF-1*基因被敲除后，导致肺发育不全，鼠死于呼吸衰竭，TTF-1对肺组织的形成和分化及保持肺的末端呼吸单位功能具有十分重要的作用。在NSCLC组织中，60%-80%的肺腺癌，0-27%的肺鳞癌及0-25%的大细胞癌表达TTF-1，以此鉴别肺腺癌和其它类型的NSCLC或者原发性肺腺癌和转移性肺腺癌。进一步的研究发现TTF-1的表达与患者的预后密切相关，TTF-1在肺腺癌的形成中所起的作用及其作用机理不清楚，且存在争议。

Winslow等^[9]^应用基因重组慢病毒载体构建鼠肺腺癌模型，构建了原发性肺腺癌无转移（TnonMet)、原发性肺腺癌有转移（Tmet）、区域淋巴结转移及远处转移（Met）的细胞系，发现在TMet和Met中缺乏TTF-1的mRNA及蛋白表达，低分化腺癌TTF-1低表达，而高分化中持续表达。本研究提示：93%宣威肺腺癌患者表达TTF-1，表达强度随着肿瘤分化程度增高而增高，高分化腺癌高表达TTF-1，与Ki-67表达负相关，两者均具有统计学意义，与张鹏等^[10]^研究一致。Fujita等^[11]^和Zu等^[6]^显示TTF-1 mRNA在NSCLC系表达频率低，包括腺癌细胞分别为50%及25%，人类肺腺癌组织多达80%有TTF-1蛋白表达，本研究宣威肺腺癌细胞株TTF-1 mRNA低表达，蛋白表达阴性，Ki-67表达100%阳性，说明肺癌细胞系细胞增殖旺盛，增殖率高，所以缺乏TTF-1的表达。

一项*meta*分析^[12]^显示TTF-1在NSCLC中高表达与患者预后好密切相关，特别是在早期或者局部晚期的腺癌。Tan等^[13]^报道一组126例NSCLC患者中TTF-1阳性表达者中位生存期超过53.2个月，而阴性表达者仅为39.4个月。本研究将96例宣威肺腺癌分为两组，TTF-1强阳性组与弱阳性和阴性组对比，中位生存有所延长（45个月*vs* 35个月），差异有统计学意义。Solis等^[14]^在肺腺癌亚组非实性腺癌组得出同样结论。刘标等^[15]^最新研究显示TTF-1高表达的贴壁为主型、乳头为主型和微乳头为主型腺癌中可能存在*EGFR*突变；而TTF-1阴性的浸润性黏液腺癌和胶样腺癌则可能存在*Kras*突变。Chung等^[16]^对496例进展期肺腺癌患者TTF-1的蛋白表达及*EGFR*突变分析，443例TTF-1阳性表达，TTF-1阳性与阴性患者比较总生存27.4个月*vs* 11.8个月，在*EGFR*突变患者中，TTF-1阳性与阴性患者比较无进展生存8.7个月*vs* 5.7个月，均有统计学意义，提示TTF-1在肺癌发生发展过程中可能起到重要作用，其表达的缺失引起肿瘤侵袭性增强，从而影响患者预后。然而，Yoon等^[17]^在79例手术的NSCLC肺癌患者外周血循环肿瘤细胞中检测TTF-1 mRNA表达发现术后TTF-1阳性的患者无进展生存明显缩短，Weir等^[18]^在528例冻存肺腺癌标本中提取DNA进行单核苷酸多态性分析显示：TTF-1在肺腺癌中是最常见的扩增子之一。这些相互矛盾的结果说明，TTF-1也许在肿瘤不同发展阶段发挥不同的作用^[9]^，主要与它的结构有关，羟基末端及羧基末端位于两端，DNA-结合同源结构域居中央，两端分别与多个转录因子结合，TTF-1与这些转录因子相互作用，并参与了多个肺癌基因的调控，能够起协同或拮抗作用，促进或抑制肺癌生长和转移。本研究样本量少，需要进一步在宣威肺腺癌中扩大样本研究。

综上所述，TTF-1在宣威肺腺癌中可能是一个肿瘤抑制基因，TTF-1的表达与病理分化程度有关，随着病理分化程度的增高而增强，与Ki-67表达负相关，并且可作为宣威肺腺癌患者预后好的指标。
